# The dietary intake of chronic kidney disease according to stages: Findings from the Korean National Health and Nutritional Examination Survey

**DOI:** 10.1371/journal.pone.0260242

**Published:** 2021-11-29

**Authors:** Seon-Mi Kim, Min-ho Kim, Dong-Ryeol Ryu, Hyung Jung Oh

**Affiliations:** 1 Department of Internal Medicine, School of Medicine, Ewha Womans University Mokdong Hospital, Seoul, Republic of Korea; 2 Ewha Institute of Convergence Medicine, Ewha Womans University Mokdong Hospital, Seoul, Republic of Korea; 3 Research Institute for Human Health Information, Ewha Womans University Mokdong Hospital, Seoul, Republic of Korea; 4 Department of Internal Medicine, School of Medicine, Ewha Womans University, Seoul, Republic of Korea; Jouf University, Kingdom of Saudi Arabia, SAUDI ARABIA

## Abstract

Appropriate dietary adjustment in patients with chronic kidney disease (CKD) is important, and nutritional guidelines recommend different dietary management depending on the CKD stage. However, there is no study, to our knowledge, of the characteristics of dietary intake according to CKD stages. We tried to assess the comparison of nutritional intake according to CKD stages. A cross-sectional study was conducted to reveal the characteristics of dietary intake among patients with CKD based on the Korean National Health and Nutritional Examination Survey between 2011 and 2014. Of 16,878 participants, we classified non-CKD (n = 14,952) and CKD (n = 1,926), which was stratified into five groups (I, II, IIIa, IIIb, and IV–V). We investigated the characteristics of dietary intake, such as energy, water, protein, fat, carbohydrate, sodium, potassium, calcium, and phosphorus, according to stage of CKD. We also explored nutritional intake according to CKD stage among patients with early CKD (stage I and II) and advanced CKD (stage IIIa, IIIb, and IV–V). Intake of majority of nutrients and energy tended to be decreased as CKD progressed. In early CKD stage, intake of energy, water, protein, fat, carbohydrate, potassium, calcium and phosphorus seemed to be statistically significant decreased as CKD progressed. In advanced CKD stage, intake of potassium and calcium seemed to be decreased as CKD progressed, but the intake of energy was about to be lower limit. Appropriate dietary education and CKD recognition are needed to improve nutritional intake depending on the CKD stage.

## Introduction

The prevalence of chronic kidney disease (CKD) is estimated to be 8–16% worldwide [1], and over 2 million people have end-stage renal disease (ESRD) [2], which is a life-threatening outcome of CKD that requires renal replacement therapy for survival. In the Republic of Korea, the prevalence of CKD in individuals aged >20 years was 8.2% [3]. CKD is also one of the most important chronic illnesses that imposes a substantial disease in an aging society [4].

CKD treatment mainly aims to preserve renal function, as renal function continues to decrease with age and renal disease progression or complications. Other than established modifiable risk factors for CKD such as hypertension, diabetes mellitus (DM), and dyslipidemia, recent studies and guidelines suggest that nutrition is an important factor in CKD progression [2]. The significance of proper diet in CKD patients was confirmed in a large retrospective cohort study: the mortality rate was significantly lower in predialysis patients who received care from a dietitian compared to those who did not [5]. Protein-energy wasting is one of the strongest predictors of mortality in patients with CKD [6, 7]. However, nutrition and dietary patterns are often neglected as a therapeutic tools for preventing and slowing CKD progression [8].

CKD is categorized into stages, with symptoms across stages. According to the Kidney Disease Improving Global Outcomes (KDIGO) guidelines, CKD is classified into stage I–V, according to the extent of albuminuria and estimated glomerular filtration rate (eGFR) [9]. Because different clinical manifestations and adverse outcomes are observed in each CKD stage, different management strategies including dietary control, are needed to prevent CKD progression. However, most studies on nutrition in CKD patients have been focused on terminal-stage CKD, or have been conducted regardless of the CKD stage [10–13].

Thus, this study examined the dietary composition of CKD patients, and investigated whether dietary intake was altered according to the CKD stage based on the Korean National Health and Nutrition Examination Survey (KNHANES) between 2011 and 2014.

## Materials and methods

### Subjects

**Fig 1:**
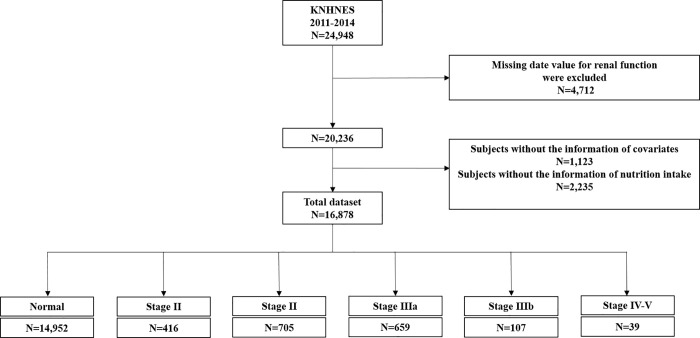
Study design and patient distribution. Abbreviations: KNHNES, Korean National Health and Nutritional Examination Survey.

KNHANES was a population-based, cross-sectional study of health and nutritional status of the non-institutionalized Korean population. The current study obtained data from the third (2005), fourth (2007–2009), fifth (2010–2012), sixth (2013–2015), and seventh (2016–2017) years of KNHANES. The Korean Center for Disease Control and Prevention (KCDC) conducted the survey using a stratified, multi-stage, clustered probability design to select a representative, nationwide sample [3]. Of subjects participating in KNHANES 2011–2014 (n = 24,948), we excluded people who did not have information about kidney function (albuminuria, serum creatinine, and eGFR; n = 4,712). Moreover, we excluded subjects who did not have information about covariates, such as comorbidities (n = 1,123), and data about dietary intake (n = 2,235). In total, 16,878 subjects were included in the final analysis, and were stratified into five groups (non-CKD, stage I, stage II, stage IIIa, stage IIIb, and CKD stage IV–V) (Fig 1).

The study was performed in accordance with the Declaration of Helsinki and was approved by the Institutional Review Board of Ewha Womans University Medical Center (EUMC 2019-06-005). All subjects provided written informed consent to participate in the study.

### Data collection

#### 1. KNHANES

The nutritional survey of KNHANES consisted of dietary behavior, 24-hour recall, and food frequency questionnaires (FFQ). Data were collected by trained dietitians, one week after a face-to-face health interview. Daily energy intake was calculated using the Korean Foods and Nutrients Database of the Rural Development Administration [14].

#### 2. Demographic and clinical characteristics

We investigated age, sex, body mass index (BMI), comorbidities such as hypertension and DM, the use of nutritional education, awareness of CKD awareness, income, occupation, residential district, and laboratory findings (serum creatinine, urinary albumin-to-creatinine ratio total cholesterol, and high-density lipoprotein).

The presence of CKD was established, based on the presence of kidney damage and level of kidney function; eGFR was calculated from standardized serum creatinine level using the Modification of Diet in Renal Disease (MDRD) equation [15]. Spot urinary albumin-to-creatinine ratio (UACR) was also calculated with mg of albumin per g of creatinine (mg/g). Based on KDIGO guidelines, we stratified CKD into five groups: stage I (eGFR ≥90 mL/min/1.73 m^2^, UACR ≥30 mg/g); stage II (eGFR 60–89 mL/min/1.73 m^2^, UACR ≥30 mg/g); stage IIIa (eGFR 45–59 mL/min/1.73 m^2^); stage IIIb (eGFR 30–44 mL/min/1.73 m^2^); and stage IV–V (eGFR <30 mL/min/1.73 m^2^). However, there were only a few patients (n = 39) in stage IV–V, so we decided to classify these two stages as one group. Further, we stratified the CKD patients into two groups: early CKD (stage I–II) versus advanced CKD (stages III–V), and explored the characteristics of dietary intake in each group [16].

Hypertension was defined as subjects with systolic blood pressure ≥140 mmHg, diastolic blood pressure ≥90 mmHg, or who had been taking antihypertensive medication. In addition, DM was defined as patients with serum fasting glucose level ≥126 mg/dL, who had been taking antidiabetic medicine, or who had a previous diagnosis of DM.

The use of nutritional education was defined as nutritional counseling conducted by a dietitian in a public health center, ward office, resident center, welfare facility, school, or hospital in the previous year.

CKD awareness was regarded as when subjects recognized their diagnosis of CKD or had been treated for CKD. Based on their monthly income, the populations were stratified into four groups: Q1, <$1,000; Q2, $1,000–$2,000; Q3, $2,000–$3,000; and Q4, >$3,000. Occupation status included three categories: participate worked for more than one hour per week, worked for more than eighteen hours here week at a family run business without pay, or between jobs.

When patients lived in one of the seven metropolitan cities (Seoul, Incheon, Daejeon, Daegu, Kwangju, Ulsan, and Busan), they were considered urban dwellers, whereas participants from other areas were considered rural dwellers.

#### 3. Statistical analysis

Baseline characteristics and laboratory findings were presented as means and standard deviations or as medians and interquartile ranges. The variables were tested for normal distribution using the Kolmogorov-Smirnov test, and then differences between stages were tested using ANOVA test (normal distribution) or the Mann-Whitney test (non-normal distribution) for continuous variables. Categorical variables were compared by χ^2^–test. Based on CKD stage, the trends of nutritional intake were compared by Spearman’s correlation. We used R (version 3.5.0, Statistical computing, Vienna, Austria) to reveal trends in nutrient intake according to CKD stage using graphical methods. Statistical analyses were conducted using SAS version 9.4 (SAS Institute Inc, Cary, NC, USA), and a p value <0.05 was considered statistically significant.

## Results

### 1. Baseline characteristics

Table 1 shows baseline characteristics of the non-CKD and CKD stage I–V groups. Of 16,878 participants, 14,952 (88.6%) were non-CKD and 1,926 (11.4%) had CKD: 416 (2.5%) were in CKD stage I, 705 (4.2%) in stage II, 659 (3.9%) in stage IIIa, 107 (0.6%) in stage IIIb, and 39 (0.2%) in stage IV–V. In the total population of 16,878, mean age was 51.4 ± 16.2 years, and 7,109 participants (42.1%) were male. In addition, 5,364 subjects (31.8%) had hypertension, and 1,921 (11.4%) had DM. Nutritional education was given to only 708 people (4.2%). Regarding laboratory findings, median value of serum creatinine, UACR, total cholesterol, and HDL levels were 0.80 mg/dL, 3.9 mg/g, 187 mg/dL, and 49.4 mg/dL respectively.

**Table 1:** Baseline characteristics.

Variables		Total (N = 16,878, 100%)	Non-CKD (N = 14,952, 88.6%)	CKD I (N = 416, 2.5%)	CKD II (N = 705, 4.2%)	CKD IIIa (N = 659, 3.9%)	CKD IIIb (N = 107, 0.6%)	CKD IV-V (N = 39, 0.2%)	P -value
**Age, years**		51.4±16.2	49.9±15.8	52.9±15.9	62.9±12.6	69.2±10.0	70.3±9.3	66.2±12.9	<0.001
**Male, n (%)**		7,109 (42.1%)	6,247 (41.8%)	151 (36.3%)	312 (44.3%)	322 (48.9%)	58 (54.2%)	19 (48.7%)	<0.001
**BMI, kg/m** ^**2**^ **, n (%)**	**BMI< 18.5**	679 (4.0%)	623 (4.2%)	24 (5.8%)	13 (1.9%)	14 (2.1%)	4 (3.7%)	1 (2.56%)	<0.001
	**18.5 ≤ BMI< 25.0**	10,749 (63.7%)	9,724 (65.1%)	214 (51.4%)	366 (52.0%)	358 (54.3%)	59 (55.1%)	28 (71.8%)	
	**25.0≤ BMI< 30.0**	4,784 (23.4%)	4062 (27.2%)	148 (35.6%)	273 (38. 8%)	256 (38.9%)	36 (33.6%)	9 (23.1%)	
	**30.0≤ BMI**	654 (3.9%)	532 (3.6%)	30 (7.2%)	52 (7.4%)	31 (4.7%)	8 (7.5%)	1 (2.6%)	
	**Missing (n, %)**	12 (0.1%)	11 (0.1%)	-	1 (0.0%)	-	-	-	
**Comorbidities**	**Hypertension (n, %)**	5,364 (31.8%)	4,092 (27.4%)	213 (51.2%)	471 (66.8%)	476 (72.2%)	84 (78.5%)	28 (71.8%)	<0.001
	**Diabetes mellitus (n, %)**	1,921 (11.4%)	1,302 (8.7%)	113 (27.2%)	233 (33.1%)	206 (31.3%)	47 (43.9%)	20 (51.3%)	<0.001
**Nutritional education**	**Yes (n, %)**	708 (4.2%)	609 (4.1%)	20 (4.8%)	37 (5.3%)	30 (4.6%)	7 (6.5%)	5 (12.8%)	0.115
	**No (n, %)**	16,119 (95.8%)	14,294 (95.9%)	396 (95.2%)	667 (94.7%)	628 (95.4%)	100 (93.5%)	34 (87.2%)	
	**Missing (n, %)**	51 (0.7%)	49 (0.3%)	-	1 (0.0%)	1 (0.0%)	-	-	
**Awareness of CKD diagnosis**	**Yes (n, %)**	41 (0.2%)	0 (0.0%)	4 (1.0%)	4 (0.6%)	11 (1.7%)	6 (5.6%)	16 (41.0%)	<0.001
	**No (n, %)**	16,837 (99.8%)	14,952 (100.0%)	412 (99.0%)	701 (99.4%)	648 (98.3%)	101 (94.4%)	23 (59.0%)	
**Residential District**	**Rural area (n, %)**	9,096 (53.9%)	7,994 (53.5%)	246 (59.1%)	396 (56.2%)	376 (57.1%)	64 (59.8%)	20 (51.3%)	0.044
	**Urban area (n, %)**	7,782 (46.1%)	6,958 (46.5%)	170 (40.9%)	309 (43.8%)	283 (42.9%)	43 (40.2%)	19 (48.7%)	
**Income**	**Q1 (n, %)**	3,930 (23.3%)	3,450 (23.1%)	119 (28.6%)	188 (26.7%)	139 (21.1%)	26 (24.3%)	8 (20.5%)	0.004
	**Q2 (n, %)**	4,284 (25.4%)	3,786 (25.3%)	110 (26.4%)	169 (24.0%)	174 (26.4%)	35 (32.7%)	10 (25.6%)	
	**Q3 (n, %)**	4,237 (25.1%)	3,764 (25.7%)	107 (25.7%)	187 (26.5%)	151 (22.9%)	19 (17.8%)	9 (23.1%)	
	**Q4 (n, %)**	4,324 (25.6%)	3,865 (25.8%)	75 (18.0%)	159 (22.6%)	188 (28.5%)	25 (23.4%)	12 (30.8%)	
	**Missing (n, %)**	103 (0.6%)	87 (0.6%)	5 (1.2%)	2 (0.2%)	7 (1.1%)	2 (1.9%)	-	
**Occupation**	**Yes (n, %)**	9,846 (58.3%)	8,995 (60.2%)	252 (60.6%)	326 (46.2%)	234 (35.5%)	24 (22.4%)	15 (38.5%)	<0.001
	**No (n, %)**	6,979 (41.3%)	5,913 (39.5%)	163 (39.2%)	377 (53.5%)	421 (63.9%)	82 (76.6%)	23 (59.0%)	
	**Missing (n, %)**	53 (0.3%)	44 (0.3%)	1 (0.0%)	2 (0.3%)	4 (0.6%)	1 (0.9%)	1 (2.6%)	
**Laboratory findings**									
**Serum creatinine, mg/dL**		0.80 (0.69–0.95)	0.79 (0.69–0.93)	0.65 (0.60–0.76)	0.86 (0.75–0.98)	1.17 (1.00–1.30)	1.58 (1.32–1.71)	2.37 (2.04–3.30)	<0.001
**Urinary albumin-to-creatinine ratio, mg/g**		3.9 (1.9, 8.7)	3.5 (1.8–6.7)	55.5 (39.1–104.5)	60.9 (40.7–125.3)	78.0 (32.5–310.7)	340.2 (107.1–1250.0)	282.0 (46.1–1185.5)	<0.001
**Total Cholesterol, mg/dL**		187 (165–212)	187 (166–212)	193 (164–219)	191 (168–218)	186 (160–209)	171 (147–195)	171 (150–197)	<0.001
**HDL, mg/dL**		49.4 (42.0–58.2)	49.6 (42.0–58.4)	49.7 (49.9–58.2)	45.8 (39.2–54.4)	43.9 (37.3–51.6)	41.1 (34.4–48.7)	41.3 (36.3–49.4)	<0.001

Data are expressed as mean ± standard deviations (normal distribution) or as median and interquartile range (non-normal distribution).

Abbreviations: CKD, chronic kidney disease; BMI, body mass index.

Definitions: 1) Hypertension: SBP ≥140 mmHg, DBP ≥90 mmHg, or subjects who had been taking anti-hypertensive medication.

2) Diabetes mellitus: Serum fasting glucose level ≥126 mg/dL, subjects who had been taking anti-diabetic medicine, or had a previous diagnosis of diabetes mellitus.

3) Nutritional education: Nutritional counseling conducted by a dietitian in a public health center, ward office, resident center, welfare facility, school, or hospital in the previous year.

4) Awareness of CKD: Diagnosed of CKD or had been treated for CKD.

5) Income: Q1; < $1000, Q2; $1000-$2,000, Q3; $2,000-$3,000, and Q4 >$3,000 based on monthly income.

6) Occupation: Subjects had been working for more than 1h per week, working for >18 h per week at a family run business without payment, or were between jobs.

7) Residential area: Subjects lived in one of the seven metropolitan cities (Seoul, Incheon, Daejeon, Daegu, Kwangju, Ulsan, and Busan) were considered urban dweller, whereas subjects from other areas were considered rural dwellers.

When subjects were stratified into five groups according to CKD stage and non-CKD, we found significant differences between groups in mean age, the proportion of males, BMI, the incidence of hypertension and DM, income, CKD awareness, occupation, residential district, and laboratory findings, while there was no significant difference in the incidence of nutritional education (Table 1).

### 2. Comparisons of nutritional intake between groups

**Fig 2:**
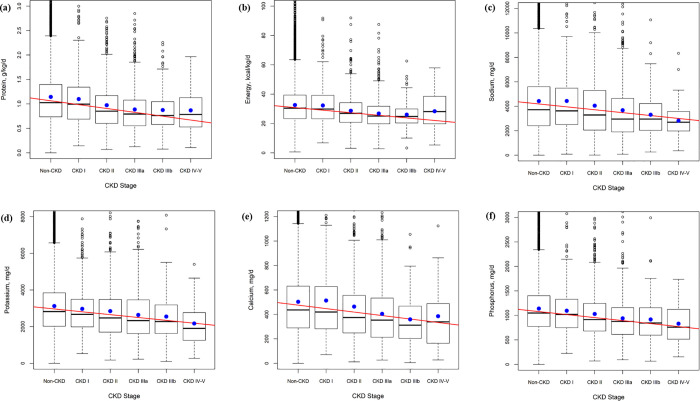
Macronutrient intake according to CKD stages. (a) Protein (b) Energy (c) Sodium (d) Potassium (e) Calcium (f) Phosphorous intake according to CKD stage.

We compared dietary intake between the groups using 24-hour recall and FFQ (Table 2). We found significant differences in dietary intake between groups: in particular, the intake of energy, water, protein, fat, carbohydrate, sodium, potassium, calcium, and phosphorus seemed to decrease as CKD progressed. Moreover, we chose six nutrients (protein, energy, sodium, potassium, calcium, and phosphorus) recommended in the KDIGO guidelines [9]. As CKD progressed, the intakes of energy, water, protein, sodium, potassium, calcium, and phosphorus tended to decrease (Fig 2).

**Table 2:** Comparisons of the nutritional intake between the groups.

Dietary constituent	Total (N = 16,878, 100%)	Non-CKD (N = 14,952, 88.4%)	CKD I (N = 416, 2.5%)	CKD II (N = 705, 4.2%)	CKD IIIa (N = 656, 3.9%)	CKD IIIb (N = 107, 0.6%)	CKD IV-V (N = 39, 0.2%)	P-value	P-value^†^
**Energy, kcal/kg/d**	30.0 (23.0–38.9)	30.5 (23.3–39.4)	29.8 (23.2–39.2)	26.9 (20.8–34.1)	25.1 (19.8–31.8)	24.8 (20.3–30.0)	28.2 (19.6–38.7)	<0.001	<0.001
**Water, g/d**	907.6 (584.7–1339.7)	929.9 (606.5–1362.7)	881.4 (572.3–1404.1)	735.0 (448.4–1123.6)	666.7 (430.0–1029.8)	687.6 (433.4–1054.3)	656.0 (335.3–1004.3)	<0.001	<0.001
**Protein, g/kg/d**	1.0 (0.7–1.4)	1.0 (0.7–1.4)	1.0 (0.7–1.3)	0.9 (0.6–1.2)	0.8 (0.6–1.1)	0.8 (0.6–1.1)	0.8 (0.5–1.1)	<0.001	<0.001
**Fat, g/d**	32.2 (18.5–52.6)	33.6 (19.7–54.0)	29.2 (17.0–49.5)	21.2 (12.6–38.5)	20.4 (10.8–33.9)	18.2 (10.1–33.8)	19.3 (9.3–39.7)	<0.001	<0.001
**Carbohydrate, g/d**	301.4 (229.7–382.6)	303.2 (231.3–384.6)	292.9 (227.5–377.5)	292.1 (225.6–372.2)	285.2 (214.0–355.0)	273.3 (215.7–352.5)	292.9 (189.4–381.1)	<0.001	<0.001
**Sodium, mg/d**	3658.0 (2372.9–5540.3)	3716.3 (2417.1–5596.3)	3624.0 (2512.1–5488.2)	3287.1 (2051.1–5284.2)	2956.6 (1904.3–4662.9)	2956.9 (2033.2–4246.3)	2694.2 (1858.7–3617.1)	<0.001	<0.001
**Potassium, mg/d**	2782.0 (1980.0–3799.3)	2822.8 (2020.6–3842.4)	2670.6 (1982.7–3490.9)	2472.3 (1701.2–3480.9)	2329.2 (1616.7–3464.3)	2278.5 (1624.0–3187.4)	1904.0 (1189.3–2841.1)	<0.001	<0.001
**Calcium, mg/d**	429.0 (283.7–625.0)	436.7 (289.3–631.4)	419.8 (282.2–628.5)	374.2 (248.4–556.2)	353.0 (213.0–534.1)	311.8 (200.6–469.5)	339.2 (157.6–495.0)	<0.001	<0.001
**Phosphorus, mg/d**	1034.4 (759.3–1384.6)	1049.2 (774.1–1401.8)	1020.7 (748.9–1329.4)	915.8 (682.0–1244.4)	876.0 (610.8–1158.8)	845.4 (583.5–1157.6)	759.1 (507.3–1148.0)	<0.001	<0.001

Data are expressed as median and interquartile range.

^**†**^ Adjusted for age and sex.

Abbreviations: CKD, chronic kidney disease.

### 3. Nutritional intake in the early CKD and advanced CKD group

We assumed that a difference in dietary intake exists between early-stage and advanced-stage CKD, so we explored nutritional intake after stratifying the population into two groups (early CKD and advanced CKD). Moreover, non-CKD subjects were included in the early CKD group, since (except for albuminuria) early-stage CKD is characterized by normal renal function. As seen in Table 3, the intakes of energy, water, protein, fat, carbohydrate, potassium, calcium and phosphorus were significantly decreased in CKD stage II versus stage I and non-CKD. In advanced CKD, the intakes of potassium and calcium were remarkably decreased as CKD progressed which was seen in Table 4.

**Table 3:** Nutritional intake in non-CKD versus early CKD group*.

Dietary constituent	Total (N = 16,073)	Non-CKD (N = 14,952, 93.0%)	CKD I (N = 416, 2.6%)	CKD II (N = 705, 4.4%)	P -value	P-value^†^
**Energy, kcal/kg/d**	30.3 (23.2–39.2)	30.5 (23.3–39.4)	29.8 (23.2–39.2)	26.9 (20.8–34.1)	< .001	< .001
**Water, g/d**	918.9 (595.7–1354.7)	929.9 (606.5–1362.7)	881.4 (572.3–1404.1)	735.0 (448.4–1123.6)	< .001	< .001
**Protein, g/kg/d**	1.0 (0.7–1.4)	1.0 (0.7–1.4)	1.0 (0.7–1.3)	0.9 (0.6–1.2)	< .001	< .001
**Fat, g/d**	32.9 (19.1–53.4)	33.6 (19.7–54.0)	29.2 (17.0–49.5)	21.2 (12.6–38.5)	< .001	< .001
**Carbohydrate, g/d**	302.5 (231.0–383.8)	303.2 (231.3–384.6)	292.9 (227.5–377.5)	292.1 (225.6–372.2)	0.024	0.005
**Sodium, mg/d**	3696.7 (2400.1–5578.3)	3716.3 (2417.1–5596.3)	3624.0 (2512.1–5488.2)	3287.1 (2051.1–5284.2)	< .001	0.317
**Potassium, mg/d**	2803.5 (2004.9–3815.0)	2822.8 (2020.6–3842.4)	2670.6 (1982.7–3490.9)	2472.3 (1701.2–3480.9)	< .001	< .001
**Calcium, mg/d**	433.2 (287.4–628.9)	436.7 (289.3–631.4)	419.8 (282.2–628.5)	374.2 (248.4–556.2)	< .001	0.001
**Phosphorus, mg/d**	1042.6 (768.8–1394.9)	1049.2 (774.1–1401.8)	1020.7 (748.9–1329.4)	915.8 (682.0–1244.4)	< .001	< .001

Data are expressed as median and interquartile range.

* Early CKD group included CKD stage I and II.

^**†**^ Adjusted for age and sex.

Abbreviations: CKD, chronic kidney disease.

**Table 4:** Nutritional intake in advanced CKD group*.

Dietary constituent	Total (N = 805)	CKD IIIa (N = 659, 81.9%)	CKD IIIb (N = 107, 13.3%)	CKD IV-V (N = 39, 4.8%)	P -value	P-value^†^
**Energy, kcal/kg/d**	25.1 (19.9–31.8)	25.1 (19.8–31.8)	24.8 (20.3–30.0)	28.2 (19.6–38.7)	0.949	0.949
**Water, g/d**	671.5 (425.4–1029.8)	666.7 (430.0–1030.0)	687.6 (433.4–1054.3)	656.0 (335.3–1004.3)	0.835	0.653
**Protein, g/kg/d**	0.8 (0.6–1.1)	0.8 (0.6–1.1)	0.8 (0.6–1.1)	0.8 (0.5–1.1)	0.933	0.801
**Fat, g/d**	19.6 (10.7–33.9)	20.4 (10.8–33.9)	18.2 (10.1–33.8)	19.3 (9.3–39.7)	0.756	0.571
**Carbohydrate, g/d**	284.4 (212.8–356.2)	285.2 (214.0–355.0)	273.3 (215.7–352.5)	292.9 (189.4–381.1)	0.533	0.369
**Sodium, mg/d**	2934.3 (1931.8–4539.0)	2956.6 (1904.3–4662.9)	2956.9 (2033.2–4246.3)	2694.2 (1858.7–3617.1)	0.188	0.103
**Potassium, mg/d**	2310.3 (1601.7–3360.2)	2329.2 (1616.7–3464.3)	2278.5 (1624.0–3187.4)	1904.0 (1189.3–2841.1)	0.103	0.045
**Calcium, mg/d**	343.4 (210.7–519.1)	353.0 (213.0–534.1)	311.8 (200.6–469.5)	339.2 (157.6–495.0)	0.068	0.032
**Phosphorus, mg/d**	863.5 (600.9–1154.4)	876.0 (610.8–1158.8)	845.4 (583.5–1157.6)	759.1 (507.3–1148.0)	0.210	0.093

Data are expressed as median and interquartile range.

* Advanced CKD group included CKD stage IIIa, IIIb, IV, and V.

^**†**^ Adjusted for age and sex, sex.

Abbreviations; CKD, chronic kidney disease.

## Discussion

Appropriate dietary intake is essential for CKD patients, to prevent or delay ESRD progression and the occurrence of CKD-related complications [13, 17]. To our knowledge, no study has investigated dietary intake according to the CKD stage. In this study, we found significant differences in dietary intake according to the CKD stage and observed different patterns of dietary intake between the early CKD and advanced CKD group.

The KDIGO guidelines suggest the need to stratify CKD based on kidney damage and function, which means that different clinical manifestations and adverse outcomes are expected in each stage, and physicians may need to take care of patients with different strategies, including dietary control, in accordance with the CKD status. Identification of the major nutrient sources can help establish effective dietary strategies targeted towards the nutritional care of CKD patients. Kidney Disease Outcomes Quality Initiative (KDOQI) guidelines recommend that CKD patients limit their intake of proteins, sodium, phosphorus, and potassium to maintain their serum nutrient levels within the normal range [18]. Dietary restriction of proteins is inevitably less than the rest of the nutrients, thereby causing nutritional imbalance. Thus, the effect of a low-protein diet remains controversial, although it proved beneficial in preventing the deterioration of renal function, it did not reduce all-cause mortality [19]. In this study, we also found that the intake of most nutrients decreases with CKD progression. Moreover, lipid profile is a potential tool to assess protein-energy wasting in CKD patients [20], and the median values of cholesterol and HDL tended to decrease with the progression of CKD stage (Table 1). Considering that the nutrient intake and lipid profile showed a declining association with CKD progression, the risk of malnutrition has become a concerning in the context of CKD.

We explored nutritional intake after stratifying the population into two groups, early CKD and advanced CKD. Early CKD is defined by an eGFR of ≥60 ml/min/1.73 m^2^ with substantial proteinuria, whereas advanced CKD is characterized by an eGFR of <60 ml/min/ 1.73 m^2^. Early CKD is free from nutritional restriction compared with advanced CKD, but it can be seen that the intake of most nutrients decreases with CKD progression. Moreover, in advanced CKD, the intake of potassium and calcium seemed to decrease with CKD progression, but the intake of energy was the approximate lower limit. Protein intake restriction is thought to play an important role in CKD progression. However, the published researches and meta-analyses on the effect of protein restriction cannot answer this question in clinical practice, such as kidney failure events, all-cause mortality, and nutritional status [19, 21, 22]. Therefore, most nephrologists recommend no restrictions or only mild restrictions on protein intake (0.8–1 g/kg daily). We showed that protein and energy intake tended to decrease as the CKD stage progressed. Adherence to a low-protein diet can be improved by ensuring adequate energy intake [23]; therefore, fat and carbohydrate content should together account for more than 90% of the daily energy intake requirement of 30–35 kcal/kg to avoid protein-energy wasting, which can be achieved by increasing the trend of energy intake with CKD progression [24]. Although the intake of carbohydrate and fat was slightly increased in the CKD IV-V, it was insufficient to maintain the total energy intake. Appropriate glycemic control is important in advanced CKD because DM prevalence increases with the progression of CKD stage (Table 1). It is therefore necessary to tailor the diet to the patient’s condition, and not just adopt a one-size-fits-all approach. Nutrient intake in CKD patients should consider the patients’ overall metabolic state and comorbid conditions.

Owing to such abovementioned concerns, nutritional interventions with disease-specific dietary ranges are needed. To address this, we focused on the CKD awareness rate. There were few participants with CKD awareness (41 patients, 2.1%) among all CKD patients (1,926 patients); moreover, only 33 patients (4.1%) in the advanced CKD group perceived their kidney problems in this study. In 1999–2000, the US National Health and Nutrition Examination Survey (NHANES) demonstrated that only 2% of the adult population self-reported recognition of a weak or failing kidney [25]. Moreover, between 1999 and 2004, NHANES surveys showed that CKD awareness rates were only 8% for CKD stage 3, but 41% for stage 4 [26]. The low proportion of CKD awareness in our study is consistent with previous studies. Although several trials have been proposed to improve CKD awareness, it is difficult to find evidence of such enhanced awareness [27]. In this study, we found that the rate of awareness not only in advanced CKD but also early CKD was lower than 1%. James *et al*. emphasized that early recognition of CKD [28], and early interventions (including dietary control), might reduce the risk of progression to kidney failure. Thus, in the future, the effectiveness of CKD awareness and changing dietary intake in CKD patients will need to be investigated in larger CKD-recognized populations. A better understanding of the renal condition is required among patients with CKD to facilitate the detection of this disease and implement therapeutic strategies for minimizing the associated complications.

Although proper nutritional education is considered a first step for adequate nutrition in CKD patients [29], only a few advanced CKD patients recognized that they had received nutritional education. Despite the higher rate of nutritional education in CKD progression, this study showed that the rate of nutritional education was generally poor. Consistent with a previous study that emphasized the importance of dietary education among predialysis patients to reduce mortality [5], more efforts are needed to provide effective education for CKD. In the Republic of Korea, insurance-covered nutritional education programs are available for CKD patients at initial diagnosis and when dialysis is started. Thus, nutritional education may have a reduced influence on altering dietary intake as CKD progresses. New educational approaches are being developed through research and quality improvement efforts to overcome the challenges of improving CKD awareness. The optimal diet for CKD patients remains controversial depending on the kidney function, type of kidney disease and the presence of other comorbidities such as DM, hypertension, or heart failure. Therefore, effective strategy to increase the effectiveness of CKD education is to provide customized education for individuals with CKD. With this approach, patients’ adherence and compliance must be considered when proper nutrients are prescribed in advanced CKD. In this study, inappropriate nutrition patterns were identified across different CKD stages, and the first step toward correcting this pattern was to enhance the extent of nutritional education and CKD awareness.

There are some limitations to this study. First, the study was designed as a cross-sectional, retrospective trial, and it was challenging to validate the causal relationship between current nutritional intake and CKD deterioration. Second, although the FFQ used in this study had been validated elsewhere, there are several limitations associated with the questionnaire alone. FFQ can be underestimated because of the inadequate coverage of all available food items consumed by an individual [30]. Moreover, dietary assessments via FFQ and 24-h recall are limiting to the determination of dietary variations. Third in this study, 805 patients (4.8%) among the total participants were in the advanced CKD group. In particular, only a small number of patients (0.2%) belonged to the CKD IV-V group. The prevalence of CKD in the current study was lower than that of CKD in the Republic of Korea [3], which might have been underestimated. Fourth, we used MDRD equation for calculating eGFR. The accuracy of the CKD-EPI equation was significantly greater than the equation used in the MDRD study for patients with an eGFR of >60 ml per minute per 1.73 m^2^ or a BMI of >30 kg per m^2^. Thus, we believe that it is better to use CKD-EPI equation for the South Korean population. Finally, when we investigated CKD awareness, this was regarded as when subjects recognized they had been diagnosed with CKD or had been treated for CKD. Thus, CKD recognition was somewhat dependent on memory of the participants. Moreover, nutritional education was also examined based on participant memory, which means that CKD awareness and nutritional education could be underestimated. Despite these limitations, this is the first study to reveal the characteristics of dietary intake according to CKD stage. To our knowledge, this is the first report to estimate the nutrient status across the CKD stages I-V. As dietary information was systematically collected, we were able to compare results among individuals at different stages of CKD and compare their dietary patterns with those of individuals without CKD.

In conclusion, we observed varying dietary intake patterns across several CKD stages and recognized some flaws in the nutritional status at each stage. Therefore, we believe that regular assessment of dietary protein, energy, and micronutrient intake is necessary. In the future, prospective longitudinal studies with larger populations are needed to corroborate the association of between CKD awareness, nutritional education, and CKD progression.

## Conclusion

We identified differences in nutritional intake among patients with CKD who were stratified according to their CKD stage. The risk of nutritional imbalance should be recognized in light of the declining trend seen for the intake rates of a majority of nutrients depending on CKD progression. In early CKD stage, the intake of energy, water, protein, fat, carbohydrate, potassium, calcium, and phosphorus seemed to significantly decrease as CKD progressed. In advanced CKD stage, the intake potassium and calcium seemed to decrease with CKD progression, but the intake of energy was about to be lower limit. Appropriate dietary education and CKD awareness is needed to improve nutritional intake across various CKD stages.

## References

[1] Hill NR, Fatoba ST, Oke JL, Hirst JA, O’Callaghan CA, Lasserson DS, et al. Global Prevalence of Chronic Kidney Disease—A Systematic Review and Meta-Analysis. PLoS One. 2016;11(7):e0158765. Epub 2016/07/08. doi: 10.1371/journal.pone.0158765 27383068 PMC4934905

[2] Mills KT, Xu Y, Zhang W, Bundy JD, Chen CS, Kelly TN, et al. A systematic analysis of worldwide population-based data on the global burden of chronic kidney disease in 2010. Kidney Int. 2015;88(5):950–7. Epub 2015/07/30. doi: 10.1038/ki.2015.230 26221752 PMC4653075

[3] Park JI, Baek H, Jung HH. Prevalence of Chronic Kidney Disease in Korea: the Korean National Health and Nutritional Examination Survey 2011–2013. J Korean Med Sci. 2016;31(6):915–23. Epub 2016/06/02. doi: 10.3346/jkms.2016.31.6.915 27247501 PMC4853671

[4] Mallappallil M, Friedman EA, Delano BG, McFarlane SI, Salifu MO. Chronic kidney disease in the elderly: evaluation and management. Clin Pract (Lond). 2014;11(5):525–35. Epub 2015/01/16. doi: 10.2217/cpr.14.46 25589951 PMC4291282

[5] Slinin Y, Guo H, Gilbertson DT, Mau LW, Ensrud K, Collins AJ, et al. Prehemodialysis care by dietitians and first-year mortality after initiation of hemodialysis. Am J Kidney Dis. 2011;58(4):583–90. Epub 2011/07/02. doi: 10.1053/j.ajkd.2011.03.032 21719177 PMC4882105

[6] Lodebo BT, Shah A, Kopple JD. Is it Important to Prevent and Treat Protein-Energy Wasting in Chronic Kidney Disease and Chronic Dialysis Patients? J Ren Nutr. 2018;28(6):369–79. Epub 2018/07/31. doi: 10.1053/j.jrn.2018.04.002 30057212

[7] Kovesdy CP, Kalantar-Zadeh K. Why is protein-energy wasting associated with mortality in chronic kidney disease? Semin Nephrol. 2009;29(1):3–14. Epub 2009/01/06. doi: 10.1016/j.semnephrol.2008.10.002 19121469 PMC5500837

[8] Snelson M, Clarke RE, Coughlan MT. Stirring the Pot: Can Dietary Modification Alleviate the Burden of CKD? Nutrients. 2017;9(3). Epub 2017/03/14. doi: 10.3390/nu9030265 28287463 PMC5372928

[9] Stevens PE, Levin A. Evaluation and management of chronic kidney disease: synopsis of the kidney disease: improving global outcomes 2012 clinical practice guideline. Ann Intern Med. 2013;158(11):825–30. Epub 2013/06/05. doi: 10.7326/0003-4819-158-11-201306040-00007 23732715

[10] Strippoli GF, Craig JC, Rochtchina E, Flood VM, Wang JJ, Mitchell P. Fluid and nutrient intake and risk of chronic kidney disease. Nephrology (Carlton). 2011;16(3):326–34. Epub 2011/02/24. doi: 10.1111/j.1440-1797.2010.01415.x 21342326

[11] Kalantar-Zadeh K, Fouque D. Nutritional Management of Chronic Kidney Disease. N Engl J Med. 2017;377(18):1765–76. Epub 2017/11/02. doi: 10.1056/NEJMra1700312 29091561

[12] Hyun YY, Lee KB, Han SH, Kim YH, Kim YS, Lee SW, et al. Nutritional Status in Adults with Predialysis Chronic Kidney Disease: KNOW-CKD Study. J Korean Med Sci. 2017;32(2):257–63. Epub 2017/01/04. doi: 10.3346/jkms.2017.32.2.257 28049236 PMC5219991

[13] Kim J, Lee J, Kim KN, Oh KH, Ahn C, Lee J, et al. Association between Dietary Mineral Intake and Chronic Kidney Disease: The Health Examinees (HEXA) Study. Int J Environ Res Public Health. 2018;15(6). Epub 2018/05/26. doi: 10.3390/ijerph15061070 29795052 PMC6025644

[14] Kweon S, Kim Y, Jang MJ, Kim Y, Kim K, Choi S, et al. Data resource profile: the Korea National Health and Nutrition Examination Survey (KNHANES). Int J Epidemiol. 2014;43(1):69–77. Epub 2014/03/04. doi: 10.1093/ije/dyt228 24585853 PMC3937975

[15] Levey AS, Coresh J, Greene T, Stevens LA, Zhang YL, Hendriksen S, et al. Using standardized serum creatinine values in the modification of diet in renal disease study equation for estimating glomerular filtration rate. Ann Intern Med. 2006;145(4):247–54. Epub 2006/08/16. doi: 10.7326/0003-4819-145-4-200608150-00004 16908915

[16] Wouters OJ, O’Donoghue DJ, Ritchie J, Kanavos PG, Narva AS. Early chronic kidney disease: diagnosis, management and models of care. Nat Rev Nephrol. 2015;11(8):491–502. Epub 2015/06/10. doi: 10.1038/nrneph.2015.85 26055354 PMC4531835

[17] Ko GJ, Kalantar-Zadeh K, Goldstein-Fuchs J, Rhee CM. Dietary Approaches in the Management of Diabetic Patients with Kidney Disease. Nutrients. 2017;9(8). Epub 2017/08/02. doi: 10.3390/nu9080824 28758978 PMC5579617

[18] Ikizler TA, Burrowes JD, Byham-Gray LD, Campbell KL, Carrero JJ, Chan W, et al. KDOQI Clinical Practice Guideline for Nutrition in CKD: 2020 Update. Am J Kidney Dis. 2020;76(3 Suppl 1):S1–s107. Epub 2020/08/25. doi: 10.1053/j.ajkd.2020.05.006 32829751

[19] Yan B, Su X, Xu B, Qiao X, Wang L. Effect of diet protein restriction on progression of chronic kidney disease: A systematic review and meta-analysis. PLoS One. 2018;13(11):e0206134. Epub 2018/11/08. doi: 10.1371/journal.pone.0206134 30403710 PMC6221301

[20] Fouque D, Kalantar-Zadeh K, Kopple J, Cano N, Chauveau P, Cuppari L, et al. A proposed nomenclature and diagnostic criteria for protein-energy wasting in acute and chronic kidney disease. Kidney Int. 2008;73(4):391–8. Epub 2007/12/21. doi: 10.1038/sj.ki.5002585 18094682

[21] Pedrini MT, Levey AS, Lau J, Chalmers TC, Wang PH. The effect of dietary protein restriction on the progression of diabetic and nondiabetic renal diseases: a meta-analysis. Ann Intern Med. 1996;124(7):627–32. doi: 10.7326/0003-4819-124-7-199604010-00002 8607590

[22] Klahr S, Levey AS, Beck GJ, Caggiula AW, Hunsicker L, Kusek JW, et al. The effects of dietary protein restriction and blood-pressure control on the progression of chronic renal disease. Modification of Diet in Renal Disease Study Group. N Engl J Med. 1994;330(13):877–84. doi: 10.1056/NEJM199403313301301 8114857

[23] Wu HL, Sung JM, Kao MD, Wang MC, Tseng CC, Chen ST. Nonprotein calorie supplement improves adherence to low-protein diet and exerts beneficial responses on renal function in chronic kidney disease. J Ren Nutr. 2013;23(4):271–6. Epub 2012/11/08. doi: 10.1053/j.jrn.2012.09.003 23131574

[24] Kovesdy CP, Kopple JD, Kalantar-Zadeh K. Management of protein-energy wasting in non-dialysis-dependent chronic kidney disease: reconciling low protein intake with nutritional therapy. Am J Clin Nutr. 2013;97(6):1163–77. Epub 2013/05/03. doi: 10.3945/ajcn.112.036418 23636234 PMC3652918

[25] Coresh J, Byrd-Holt D, Astor BC, Briggs JP, Eggers PW, Lacher DA, et al. Chronic kidney disease awareness, prevalence, and trends among U.S. adults, 1999 to 2000. J Am Soc Nephrol. 2005;16(1):180–8. Epub 2004/11/26. doi: 10.1681/ASN.2004070539 . 15563563

[26] Plantinga LC, Boulware LE, Coresh J, Stevens LA, Miller ER, 3rd, Saran R, et al. Patient awareness of chronic kidney disease: trends and predictors. Arch Intern Med. 2008;168(20):2268–75. Epub 2008/11/13. doi: 10.1001/archinte.168.20.2268 ; PubMed Central PMCID: PMC2652122. 19001205 PMC2652122

[27] Tuot DS, Plantinga LC, Hsu CY, Jordan R, Burrows NR, Hedgeman E, et al. Chronic kidney disease awareness among individuals with clinical markers of kidney dysfunction. Clin J Am Soc Nephrol. 2011;6(8):1838–44. Epub 2011/07/26. doi: 10.2215/CJN.00730111 ; PubMed Central PMCID: PMC3156423. 21784832 PMC3156423

[28] James MT, Hemmelgarn BR, Tonelli M. Early recognition and prevention of chronic kidney disease. Lancet. 2010;375(9722):1296–309. Epub 2010/04/13. doi: 10.1016/S0140-6736(09)62004-3 . 20382326

[29] Ash S, Campbell KL, Bogard J, Millichamp A. Nutrition prescription to achieve positive outcomes in chronic kidney disease: a systematic review. Nutrients. 2014;6(1):416–51. Epub 2014/01/24. doi: 10.3390/nu6010416 ; PubMed Central PMCID: PMC3916870. 24451311 PMC3916870

[30] Shim JS, Oh K, Kim HC. Dietary assessment methods in epidemiologic studies. Epidemiol Health. 2014;36:e2014009. Epub 2014/08/01. doi: 10.4178/epih/e2014009 ; PubMed Central PMCID: PMC4154347. 25078382 PMC4154347

